# Hygiene may attenuate selection for antibiotic resistance by changing microbial community structure

**DOI:** 10.1093/emph/eoac038

**Published:** 2023-01-18

**Authors:** Magnus Aspenberg, Sara Maad Sasane, Fredrik Nilsson, Sam P Brown, Kristofer Wollein Waldetoft

**Affiliations:** Centre for Mathematical Sciences, Lund University, Lund, Sweden; Centre for Mathematical Sciences, Lund University, Lund, Sweden; Department of Clinical Pharmacology, Lund University Hospital, Lund, Sweden; School of Biological Sciences, Georgia Institute of Technology, Atlanta, GA, USA; Center for Microbial Dynamics and Infection, GeorgiaInstitute of Technology, Atlanta, GA, USA; School of Biological Sciences, Georgia Institute of Technology, Atlanta, GA, USA; Center for Microbial Dynamics and Infection, GeorgiaInstitute of Technology, Atlanta, GA, USA; Torsby Hospital, Torsby, Sweden

**Keywords:** antibiotic resistance, hygiene, competitive release, ecology, metacommunity ecology

## Abstract

Good hygiene, in both health care and the community, is central to containing the rise of antibiotic resistance, as well as to infection control more generally. But despite the well-known importance, the ecological mechanisms by which hygiene (or other transmission control measures) affect the evolution of resistance remain to be elucidated. Using metacommunity ecology theory, we here propose that hygiene attenuates the effect of antibiotic selection pressure. Specifically, we predict that hygiene limits the scope for antibiotics to induce competitive release of resistant bacteria within treated hosts, and that this is due to an effect of hygiene on the distribution of resistant and sensitive strains in the host population. We show this in a mathematical model of bacterial metacommunity dynamics, and test the results against data on antibiotic resistance, antibiotic treatment, and the use of alcohol-based hand rub in long-term care facilities. The data are consistent with hand rub use attenuating the resistance promoting effect of antibiotic treatment. Our results underscore the importance of hygiene, and point to a concrete way to weaken the link between antibiotic use and increasing resistance.

## Teaser

Hygiene and antibiotic use are important levers in the work to limit resistance evolution. We study how they interact, and find that hygiene can attenuate the effect of antibiotic pressure on the rate of evolution.

## INTRODUCTION

Antibiotics have revolutionized modern medicine, but they also drive the evolution of resistance, and thereby contribute to their own demise [1, 2]. To meet this challenge, there is intense work in both evolutionary theory and clinical practice to limit or optimize antibiotic use [3–5]. A key task is to manage collateral antibiotic exposure of the commensal microbiota, of which medically important bacteria are often a part. Indeed, a recent analysis found that such ‘bystander selection’ dominates antibiotic exposure for common human pathogens [6].

In addition to the role of antibiotic use, there is within the medical profession a firm appreciation of the importance of hygiene in the management of antibiotic resistance. Measures of hygiene, such as hand disinfection, are fundamental to good medical practice after the discoveries of Semmelweis Ignác Fülöp in the mid 1800s [7], and form an integral part of current efforts to contain antibiotic resistant bacteria [8]. Moreover, general hygiene and sanitation in the community are considered important in slowing the evolution of resistance, with the rationale that they limit antibiotic consumption by decreasing the incidence of infection [9]. In this paper, we use ‘hygiene’ to denote measures to limit microbial transmission among individuals, such as hand disinfection in health care settings.

It is thus recognized that antibiotic use and hygiene are key factors in the evolution of resistance, and that this evolution often takes place in a microbial community—but these insights have yet to be put together. This is the task of the present investigation: to study the joint effects of hygiene and antibiotic use on the rate of increase of resistant bacteria in microbial communities, where these communities are commensal, and the antibiotic exposure is collateral. It requires that we integrate the within-host ecological effects of antibiotics with the between-host process of bacterial dispersal, where the latter is affected by hygiene, and possibly other factors, such as population density or cultural habits. This sits most naturally within the theoretical framework of metacommunity ecology [10, 11], and we will frame both informal discussion and mathematical analysis using this approach. However, since metacommunity theory is new to antibiotic resistance studies, we also reproduce the key result within the epidemiological compartmental modelling framework that is currently standard in the field [12] (see Supplementary A for details).

The aim of this paper is to introduce the hypothesis that hygiene attenuates the effect of antibiotic pressure on resistance evolution, and address its plausibility. The paper has three parts. First, we informally explain how metacommunity theory predicts that hygiene should limit the within-host ecological response to antibiotic treatment. Second, we develop a mathematical model of resistance dynamics in a metacommunity context and show that this prediction obtains in the model. Third, we test the prediction against antibiotic resistance data from the European Centre for Disease Prevention and Control (ECDC) and find that it is consistent with the data.

### Metacommunity ecology implies that hygiene should attenuate competitive release

Bacteria form local communities within individual hosts, and they transmit between host individuals. In ecological terms, bacteria thus form metacommunities, that is, networks of local communities interconnected by dispersal [13]. Within each local community different bacterial strains compete with each other, such that the growth and abundance of a focal strain is limited by other strains. If the local community contains a mixture of antibiotic resistant and sensitive strains, antibiotic administration is expected to kill sensitive bacteria, and relieve resistant ones of competition, allowing them to proliferate. This is an ecological phenomenon known as competitive release [14–16]. Previous work has indicated that it promotes the evolution of resistance, and that its strength depends on the dosage of the drug [17, 18].

**Figure 1.**
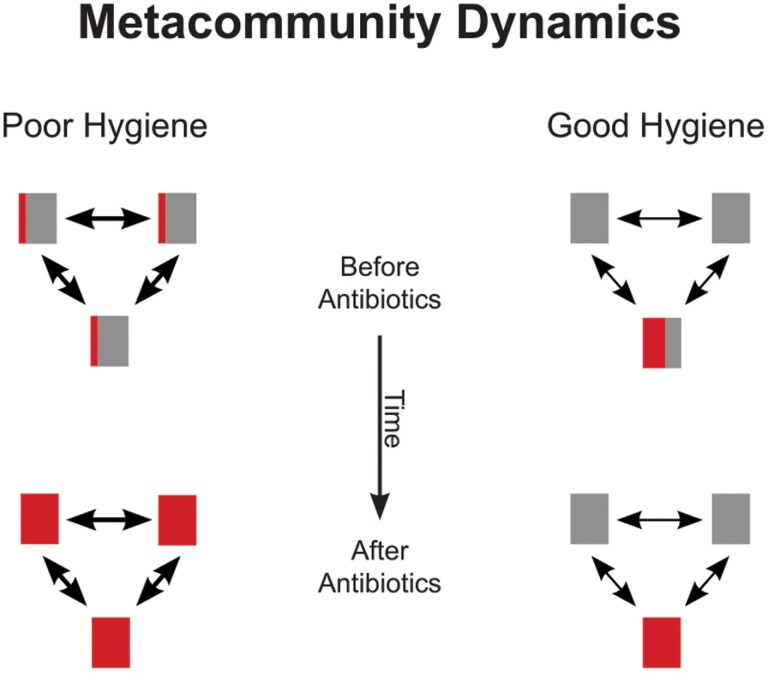
Schematic of metacommunity dynamics. Metacommunities with three host individuals are depicted. Before antibiotic treatment, resistant (red) and sensitive (grey) bacteria have similar total abundances in both poor and good hygiene settings, but they are differently distributed across host individuals. After antibiotic treatment and competitive release, resistant bacteria are more abundant in the poor hygiene setting. Red bars represent resistant strains, and grey bars sensitive strains, the area of each bar representing the abundance of the corresponding strain. Double arrows denote transmission of bacteria between hosts, thicker arrows indicating a higher rate of transmission.

However, the drug dose is only part of the picture. Competitive release of resistant bacteria requires that both sensitive and resistant strains are simultaneously present in the bacterial community within the treated host individual. And if they are, the magnitude of the release depends on the extent to which resistant strains are limited by competition from sensitive strains (see [16]). If resistant strains are absent within a host, they cannot increase (save for the possibility of de novo mutation). And, conversely, if they dominate the community already in the absence of antibiotics, they will gain little from the killing of the few sensitive cells that are present. In contrast, if resistant bacteria constitute but a small proportion of the competing community, the killing of the sensitive majority can decrease competition to a larger extent and result in a larger amplification of resistance (Figure 1).

As a consequence, the scope for competitive release is maximized when resistant strains are present in a large proportion of host individuals, and have a low abundance in the local communities in which they are present. Hence, for a given total abundance of resistant bacteria in the metacommunity, the competitive release will be larger when the resistant bacteria are more uniformly distributed across the host population.

The distribution of organisms in metacommunities is a focus of research in metacommunity ecology, and a picture has emerged that, perhaps unsurprisingly, dispersal of organisms among habitat patches tends to spread them across these patches, making them more uniformly distributed [13]. Conversely, this means that interventions that prevent dispersal should make the distribution of organisms in the metacommunity less uniform than would otherwise have been the case, and tend to isolate different types of organism in different habitat patches.

In the context of humans carrying resistant and sensitive bacteria, measures of hygiene are precisely such interventions. They should therefore make the distribution of resistant strains less uniform, leaving them abundant in some individuals and absent from others, and thus decrease the scope for competitive release.

This leads to our prediction: Increasing the level of hygiene should decrease the effect of antibiotic pressure on the competitive release of resistant bacteria. That is to say that, whilst the magnitude of competitive release increases with antibiotic pressure, this increase is less steep when the level of hygiene is higher. We focus on hygiene, because it is readily actionable and its importance is well-established, but the prediction applies to any factor that limits bacterial dispersal among host individuals.

### Mathematical modelling of bacterial metacommunity dynamics supports the prediction

In the previous section, we informally explained why metacommunity ecology theory predicts that hygiene should attenuate competitive release, in the commensal bacterial community collaterally exposed to antibiotics. We used a metacommunity framing, because it provides an intuitive and conceptually straight forward way to represent the biology involved, naturally integrating, as it is, the within- and between host levels of the system. Unfortunately, however, intuition, useful as it may be in generating hypotheses, is not a reliable guide to logical validity. Next, we therefore test this validity by subjecting the argument to formal modelling. In the main text, we use a metacommunity model, and in Supplementary A, we complement the analysis with an epidemiological compartmental model.

There are several reasons for this choice. Firstly, we wish to keep the formal analysis close to the informal argument. Secondly, we think that, at least to some extent, the benefits of the metacommunity framework for informal reasoning, such as the within- and between host level integration, translate to the formal modelling as well. Thirdly, despite the frankly obvious fact that our microbiomes form metacommunities, metacommunity theory has not yet found its way into our field. We think this is a missed opportunity, and wish to introduce this tool to the tool box of antibiotic resistance research. But promising as the metacommunity approach may be, as with other approaches, there may be peculiarities to it that affect the results, casting doubt over them. To alleviate this problem, we test our argument against a compartmental model as well. The weakness of this type of model is that its representation of the within host level is crude, but the strengths are that it is well established in the field and that it is very different from the metacommunity model, and therefore unlikely to share unfortunate peculiarities with it. We thus think that this combination is a good choice for the task at hand.

Our model of bacterial metacommunity dynamics is an extension of Hubbell’s neutral model of biogeography and relative species abundance [19, 20]. The bacterial metacommunity is modelled as a set of interconnected bacterial communities in different host individuals. In these communities there are two types of bacteria—antibiotic resistant and antibiotic sensitive—and the total number of bacteria (resistant and sensitive) in each host individual is constant, as is the number of host individuals. These constants are denoted by *J* for the number of bacteria in each host and *N* for the number of host individuals. The constant community size means that the resistant and sensitive types compete with each other, and a decrease in one entails an equal increase in the other, representing competitive release.

The model tracks the proportion of bacterial cells that are resistant in the metacommunity, as time, step by step, unfolds. In each time step bacterial cells die. In the absence of antibiotics, one cell dies in each host individual, and the resistance status of a cell does not affect its probability of dying. With increasing antibiotic pressure, however, more cells can die in each time step, and the death process becomes increasingly biased towards sensitive cells (see Supplementary A for details). We expand on this below.

Once those cells have died, they are replaced by new cells by division. These cells may originate from within the same host or from different host individuals. The probability that a new cell comes from a different host depends on the level of hygiene – the better the hygiene, the lower the probability that a bacterium transmits from another host. If the new cell originates from a different host, the probability that it is resistant is equal to the proportion of cells that are resistant in the metacommunity as a whole, and if it originates from within the focal host, the probability of resistance equals the proportion of cells that are resistant in that host. Let us compute the probabilities more precisely. Let *R*_*j*_(*t*) be the number of resistant bacteria at a given time *t* in host *j*. Since we are only looking at the transition between two adjacent time steps, we will drop the index *t* and only write *R*_*j*_.

Each time step has two phases. In the first phase one or several bacteria are lost, and in the second phase these bacteria are replaced. In the first phase, given *R*_*j*_ number of resistant bacteria in an individual, the probability that a resistant bacterium dies is


$$\begin{array}{l} P_{r}=\frac{R_{j}}{aJ}, \end{array}$$

where *a* ≥ 1 is the antibiotic pressure, represented as the differential survival of resistant and sensitive cells. Hence, *a* = 1 means that no antibiotics are present, and the probability that it is a resistant (or a sensitive) cell that dies is simply proportional to their number, whereas *a* > 1 means that antibiotic treatment makes the death process biased towards sensitive cells.

The probability that sensitive bacteria are lost is


$$\begin{array}{l} P_{s}=1-\;P_{r}. \end{array}$$

The number of sensitive bacteria lost is an integer function *ρ*(*R*, *a*) that depends on the antibiotic pressure *a* and the abundance *R* = *R*_*j*_ of resistant bacteria in host *j* (or equivalently, the abundance of sensitive bacteria *J − R*). However, in the computations (see Supplementary A), we consider *ρ* to be a continuous function that is differentiable in the *a*-direction. Moreover, we require that it is a decreasing function of *R*, meaning that more sensitive bacteria die if there are many such bacteria (i.e., a relaxation of the common assumption of a per capita death rate). We then take the integer part of this function to go back to the integer valued *ρ*. Let us denote by *µ*(*R*, *a*) the number of bacteria lost in this first phase (i.e., *µ*(*R*, *a*) = 1 or *µ*(*R*, *a*) = *ρ*(*R*, *a*)).

In the second phase, given *R* number of resistant bacteria, the probability that one resistant or one sensitive bacterium reappears is, respectively,


$$\begin{array}{l} Q_{r}=Q_{r}\left(R_{j}\right)=m\bar{s}+\left(1-m\right)s_{j},\;Q_{s}=\;1\;-\;Q_{r}, \end{array}$$ (1)

where


$$\begin{array}{l} s_{j}=\frac{R_{j}}{J-\mu \left(R_{j},a\right)}\;\;and\;\bar{s}=\frac{1}{N}\;\sum_{j=1}^{N}\frac{R_{j}}{J-\mu \left(R_{j},a\right)}, \end{array}$$ (2)

and *m* ∈ [0, 1] (called the migration rate in the literature) is the probability that a bacterium is chosen from the metacommunity as a whole, rather than the focal individual. The hygiene parameter *h* = 1 − *m*, so that good hygiene means high *h*-values and low *m*-values (and vice versa). This procedure is performed precisely *µ*(*R*, *a*) times to replace all bacteria lost in the first phase.

The time steps can be made very small in the mathematical model, and therefore we assume that the portion *µ*(*R*, *a*)/*J* is very small, although the number *µ*(*R*, *a*) can be large (in particular much larger than 1). This means that we can replace *R/(J−µ(R, a))* by *R/J* with a very small error. With this simplification, which is of minor importance (see Supplementary A), the new probabilities *Q*_*r*_ and *Q*_*s*_ then become


$$\begin{array}{l} Q_{r}=m\bar{r}+\left(1-m\right)r_{j},\;and\;Q_{s}=1-Q_{r}, \end{array}$$

where *r*_*j*_ = *R*_*j*_/*J* is the portion of resistant bacteria in host individual *j*, and $\bar{r}$ the average over all *r*_*j*_, i.e.,


$$\begin{array}{l} \bar{r}=\frac{1}{N}\sum_{j=1}^{N}\frac{R_{j}}{J}. \end{array}$$

We compute the expected change of the total number of resistant bacteria in the metacommunity in one time step. This expectation is denoted by *Z* = *Z*(*a*, *h*), which is a function of *a* and *h*. We prove that


$$\begin{array}{l} \frac{\partial^{2}Z}{\partial h\partial a}⩽0, \end{array}$$

if *a* ≥ 1, with strict inequality if *a* > 1.

Hence, the effect of antibiotic pressure on the rate of increase of resistance (∂Z/∂a) decreases with improving hygiene.

We also show that this is due to the effect of hygiene on the β-diversity of the metacommunity (see Supplementary A for details). The β-diversity is the standard deviation of the number of resistant bacteria *R*_j_ across host individuals.

### Resistance data are consistent with the prediction

In the preceding sections, we saw, first, that informal theory predicts that hygiene attenuates the effect of antibiotics on the competitive release—and thus the rate of increase—of resistant bacteria, and, second, that formal modelling supports the validity of that argument. Now, we test this prediction empirically. To do so, we restate it in statistical form.

The prediction is as follows: If a measure of the increase of resistant bacteria in a given setting is regressed upon a measure of antibiotic pressure (*a*) and a measure of hygiene (*h*) in that setting, there should be an interaction (*a* · *h*), and this should be negative. The reason is that the interaction term represents the effect that the value of one variable (e.g. *h*) has on the effect of the other variable (e.g. *a*), a negative term meaning attenuation.

We tested this prediction against data on resistance to third generation cephalosporins (a type of β-lactam antibiotics) in Enterobacteriaceae (which includes *E. coli*), the use of β-lactam antibiotics, and the use of alcohol-based hand rub in long-term care facilities in different European countries, reported by the European Centre for Disease Prevention and Control (ECDC). The merit of this data set is that it pertains to facilities where individuals stay for an extended period of time and contains information on antibiotic resistance, antibiotic use, and a quantitative measure of hygiene. The principal shortcoming is that it is cross sectional, and thus only provides the level of resistance at a given time, not the rate of increase. We therefore modelled the increase in resistance as the enrichment of resistant strains in the long-term care facilities in each country as compared with resistance in *E. coli* in the general society in that country, the rationale being that this enrichment should be a proxy for the average rate of change in resistance in the individuals in the facilities after they arrived there (see Supplementary B for details on the data and analysis).

**Figure 2.**
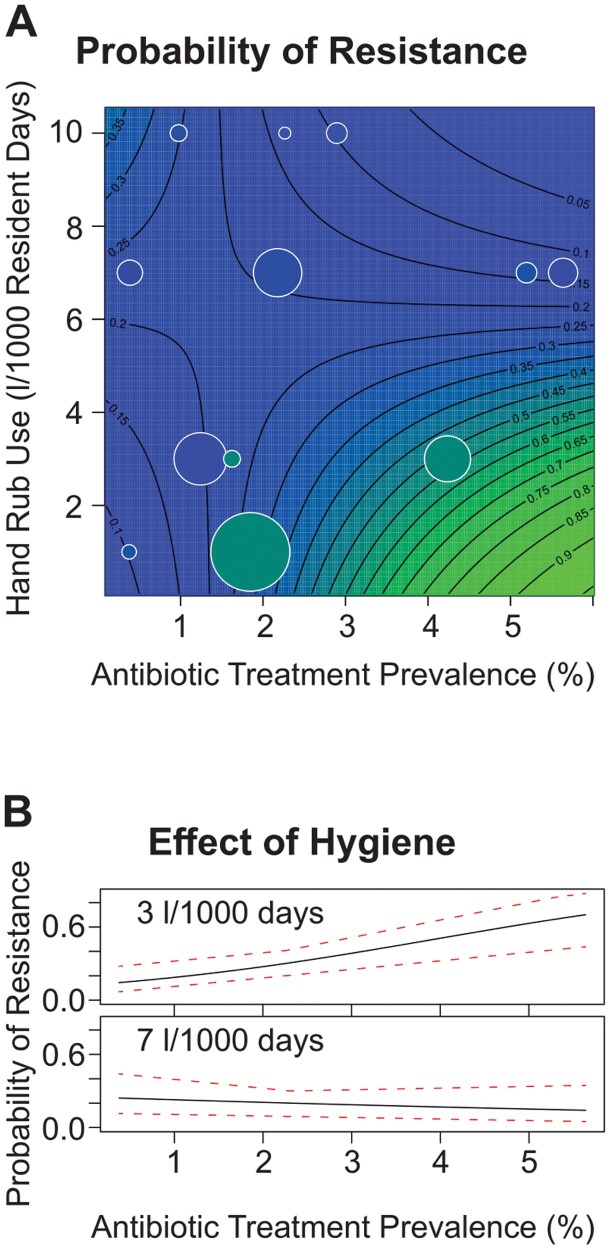
(**A**) Resistance data and statistical model. The ECDC resistance data are overlain a contour plot of the logistic regression model that was fitted to them. The probability that an Enterobacteriaceae isolate is resistant to third generation cephalosporins is plotted against the consumption of alcohol-based hand rub (vertical) and the point prevalence of β-lactam treatment (horizontal). The colour and contours represent the probability of resistance. Bubbles represent data reported for individual countries, the area being scaled to the number of bacterial isolates tested (see Supplementary B for details). (B). The effect of a moderate improvement in hygiene. Based on the data and statistical analysis given in (A), the probability that an isolate is resistant (vertical) is plotted against the prevalence of β-lactam treatment (horizontal). The upper and lower panels show this relationship for a hand rub consumption of 3 and 7 L, respectively, per 1000 resident days. Red dotted lines represent 95% confidence bands. (see Supplementary B for details).

The data were analysed by logistic regression (using R ver. 3.5.0, see [21]). In accordance with the prediction, there was a significant negative interaction between hygiene and antibiotic pressure, and this amounted to a pronounced effect of hygiene on the slope of resistance on antibiotic pressure. (The odds of resistance changes by –0.157 (CI95: –0.273 to –0.0466) per litre of hand rub per 1000 resident days and proportion of residents under β-lactam treatment; *χ*2 = 7.90, *df* = 1, *p* = 0.005.) The data and the regression model are shown in Fig. 2A, and the change in the relationship between antibiotic pressure and antibiotic resistance associated with an increase from medium-low to medium-high consumption of hand rub is illustrated in Fig. 2B.

## DISCUSSION

Here we have employed metacommunity ecology theory to study what are arguably the two most important levers available to limit the rise of resistance—the amount of antibiotics used and the level of hygiene. We found that informal theory, formal modelling, and data from the ECDC all yield the same conclusion: Improvements in hygiene weaken the link between antibiotic use and increasing resistance. The mechanism is that hygiene limits bacterial dispersal, and thereby alters the diversity that is the substrate for antibiotic induced competitive release. Since a quantitative assessment of this effect in the mathematical model requires biological information that is currently unavailable, we assessed the effect size directly with the ECDC resistance data, and found that it was quite striking (Fig. 2B).

Naturally, there are caveats to the conclusion. The data set is relatively small and pertains to a particular setting—long-term care facilities. In addition, the frequency of resistance in these facilities as compared to the baseline frequency in each country, though a measure of enrichment of resistant bacteria, is not strictly a rate of increase. Furthermore, the results may be confounded by factors not included in the analysis. For example, since each data point represents a different country, there may be differences in health care systems or cultural habits that affect the outcome. Thus, although the data lend support to the conclusion, this support is weak.

On the other hand, the validity of the argument is supported by the fact that a simple compartmental model—which is mathematically very different from the metacommunity model—whilst unable to capture the nuances of bacterial diversity, does indicate the same qualitative conclusion (see Supplementary A for details.) It is important to keep in mind, however, that both our models are designed specifically to test the validity of the informal argument, and as such, are far from comprehensive models of resistance evolution. For example, we do not include costs of resistance and related matters of potential equilibria of resistant-sensitive coexistence. We also ignored de novo mutation. From the perspective of a focal host, mutation is analogous to transmission in that it is an arrival process, whereby resistant cells are introduced to the local population and made available for competitive release. We therefore expect that it should interact with antibiotic pressure in a similar way, and that very high levels of resistance mutation would limit the scope for hygiene to inhibit resistance evolution.

In addition to our analyses, there are previous empirical results that are suggestive of a role for bacterial dispersal in modulating the effect of antibiotic pressure. Bruinsma et al. [22] investigated the resistance to several antibiotics in *E. coli* and Enterococci in the faeces of healthy volunteers living in cities with different population densities, and found that it correlated poorly with the antibiotic pressure per individual, but well with the antibiotic pressure per land area. Assuming that bacterial dispersal among individuals is facilitated by a high population density, this fits well with a modulating effect of dispersal, since the antibiotic pressure per land area is the product of the pressure per individual and the population density, and thus corresponds to the interaction term, *a* · *h*, in the statistical model above.

Whilst decreasing the population density may not be a viable route to limiting antibiotic resistance, improving health care hygiene is. Proper hand hygiene in clinical work is a cornerstone of patient safety [23], but more than 150 years after Semmelweis compliance is still poor [24]. This is unfortunate, because hand hygiene stands out among possible antiresistance interventions in that it is simple, safe, and cheap, and should therefore be possible to implement rapidly, and without major issues. Furthermore, as suggested by the findings of Bruinsma et al. [22]. above, the effect of dispersal limitation on competitive release should not be confined to the health care setting, but apply to the general community as well.

In conclusion, we have here introduced metacommunity ecology theory to the study of antibiotic resistance, and found that interventions to limit microbial dispersal may provide a means to attenuate the effect of antibiotic selection pressure on the rise of resistance.

## Supplementary Material

eoac038_suppl_Supplementary_Material

## Data Availability

The data underlying Figure 2 are available in reports from the European Centre for Disease Prevention and Control [25, 26] (see Supplementary B for details). Code availability statement. Custom code was not used.

## References

[1] Bell BG , Schellevis F, Stobberingh E et al. A systematic review and meta-analysis of the effects of antibiotic consumption on antibiotic resistance. BMC Infect Dis Jan 2014;14:13. 24405683 10.1186/1471-2334-14-13 PMC3897982

[2] Costelloe C , Metcalfe C, Lovering A et al. Effect of antibiotic prescribing in primary care on antimicrobial resistance in individual patients: systematic review and meta-analysis. BMJ 2010;340:c2096–c2096. 20483949 10.1136/bmj.c2096

[3] King LM , Fleming-Dutra KE, Hicks LA. Advances in optimizing the prescription of antibiotics in outpatient settings. BMJ 2018;363:k3047. doi:10.1136/bmj.k3047. 30420401 PMC6511972

[4] Baym M , Stone LK, Kishony R. Multidrug evolutionary strategies to reverse antibiotic resistance. Science 6268;351:2016. 10.1126/science.aad3292 PMC5496981 26722002

[5] McAdams D , Wollein Waldetoft K, Tedijanto C et al. Resistance diagnostics as a public health tool to combat antibiotic resistance: A model-based evaluation. PLoS Biol 05 2019;17:1–18. 10.1371/journal.pbio.3000250 PMC6522007 31095567

[6] Tedijanto C , Olesen SW, Grad YH et al. Estimating the proportion of bystander selection for antibiotic resistance among potentially pathogenic bacterial flora. Proc Natl Acad Sci USA 2018;115:E11988–95. 30559213 10.1073/pnas.1810840115 PMC6304942

[7] Pittet D , Boyce JM. Hand hygiene and patient care: pursuing the Sem-melweis legacy. The Lancet Infectious Diseases 2001;1:9–20. Preview Issue.11871420

[8] WHO, SAVE LIVES: clean Your Hands 5 May 2017: fight antibiotic resistance its in your hands. 2017.

[9] O’Neill J. Infection prevention, control and surveillance: limiting the development and spread of drug resistance. The Rev Antimicrob Resist March 2016. https://amr-review.org/sites/default/files/Health%20infrastructure%20and%20surveillance%20final%20version_LR_NO%20CROPS.pdf

[10] Leibold MA and Chase JM. Metacommunity Ecology (MPB-59). Princeton University Press, 2018.

[11] Mihaljevic JR. Linking metacommunity theory and symbiont evolutionary ecology. Trends Ecol Evol 2012;27:323–9. 22341499 10.1016/j.tree.2012.01.011

[12] Blanquart F. Evolutionary epidemiology models to predict the dynamics of antibiotic resistance. Evol Appl 2019;12:365–83. doi: 10.1111/eva.12753. 30828361 PMC6383707

[13] Vellend M. The Theory of Ecological Communities (MPB-57), vol. 75. Princeton University Press, 2016.

[14] Day T , Huijben S, Read AF. Is selection relevant in the evolutionary emergence of drug resistance?. Trends Microbiol 2015;23:126–33. 25680587 10.1016/j.tim.2015.01.005 PMC4494118

[15] Hansen E , Woods RJ, Read AF. How to use a chemotherapeutic agent when resistance to it threatens the patient. PLoS Biol 2017;15:1–21. 10.1371/journal.pbio.2001110 PMC5300106 28182734

[16] Wargo AR , Huijben S, de Roode JC et al. Competitive release and facilitation of drug-resistant parasites after therapeutic chemotherapy in a rodent malaria model. Proc Natl Acad Sci USA 2007;104:19914–9. 18056635 10.1073/pnas.0707766104 PMC2148397

[17] Day T , Read AF. Does high-dose antimicrobial chemotherapy prevent the evolution of resistance?. PLoS Comput Biol 2016;12:1–20. 10.1371/journal.pcbi.1004689 PMC4731197 26820986

[18] Huijben S , Bell AS, Sim DG et al. Aggressive chemotherapy and the selection of drug resistant pathogens. PLoS Pathog 2013;9:1–9. 10.1371/journal.ppat.1003578 PMC3771897 24068922

[19] Hubbell SP. A unified theory of biogeography and relative species abundance and its application to tropical rain forests and coral reefs. Coral Reefs Jun 1997;16:S9–S21.

[20] Hubbell SP. The Unified Neutral Theory of Biodiversity and Biogeography (MPB-32). Princeton University Press, 2001.

[21] R. Core Team. R: A Language and Environment for Statistical Computing. R Foundation for Statistical Computing, Vienna, Austria. https://www.R-project.org/, 2018.

[22] Bruinsma N , Hutchinson J, van den Bogaard A et al. Influence of population density on antibiotic resistance. J Antimicrob Chemother 2003;51:385–90. 12562707 10.1093/jac/dkg072

[23] WHO, WHO guidelines on hand hygiene in health care: first global patient safety challenge: clean care is safer care. 2009. 23805438

[24] Erasmus V , Daha TJ, Brug H et al. Systematic review of studies on compliance with hand hygiene guidelines in hospital care. Infect Control Hosp Epidemiol 2010;31:283294. 10.1086/650451 20088678

[25] European Centre for Disease Prevention and Control (ECDC). Point prevalence survey of healthcare-associated infections and antimicrobial use in European long-term care facilities. April-May 2013. 2014.

[26] European Centre for Disease Prevention and Control (ECDC). Antimicrobial resistance surveillance in Europe 2014. 2015.

