# Supplementary material for: Hygiene may attenuate selection for antibiotic resistance by changing microbial community structure
Source: Evol Med Public Health. 2023 Jan 18;11(1):1–7. doi: 10.1093/emph/eoac038 (PMC9847546; doi:10.1093/emph/eoac038)
Supplement: eoac038_suppl_Supplementary_Material [file eoac038_suppl_supplementary_material.pdf]

Supplement A:
The mathematical models

Analysis of the metacommunity model

1. Introduction

The mathematical model of the interaction between antibiotic pressure and hygiene is based on an idea by S. Hubbell (1). Let us here explain the basic mechanics in our model. Let N be the number of individuals in the host population, J the total number of resistant and sensitive bacteria in each individual host, m a hygiene parameter, and a a measure of the antibiotic pressure. In each time step, there are two phases. In the first phase, each host individual either loses a resistant bacterium or a number $\rho \geq 1$ of sensitive bacteria. In the second phase the individual gains the same number of bacteria of possibly different types (sensitive or resistant). Hence, after each time step, the total number of bacteria is unchanged. Let R_j be the number of resistant bacteria at some given time for a given host individual (indexed by j), and $\bar{R}$ the average number of resistant bacteria over the whole metacommunity; *i.e.*,

$$\bar{R} = \frac{1}{N}\sum_{j=1}^{N} R_j.$$

We then have the possibilities that the change of the number of resistant bacteria for an individual can attain the values $\{-1, 0, \ldots, \rho\}$ after these two phases have taken place.

The probability that a resistant bacterium is lost, given $R = R_j$ number of resistant bacteria in host j, is

$$P_r = P_r(R) = \frac{R}{aJ},$$

where a is the antibiotic pressure ($a = 1$ means that there are no antibiotics).

The probability that ρ sensitive bacteria are lost is

$$P_s = P_s(R) = 1 - P_r = 1 - \frac{R}{aJ}.$$

Let us briefly describe the properties of $\rho = \rho(x, a)$, which is a function of the antibiotic pressure a and the absolute abundance x of resistant bacteria (or equivalently, the absolute abundance of sensitive bacteria $J - x$). We may also consider ρ as a function of the relative abundance of x or $J - x$ since the total number of bacteria in each host is constant after each time step. We require that ρ is decreasing in x, meaning that more sensitive bacteria die if there are many such bacteria. We will also need a technical condition on ρ including its a-derivative (written in the Proposition below). This condition is discussed after the Proposition, and there are many "natural" functions ρ that satisfy it (for instance ρ must increase with higher a-values). Let us denote by $\mu(x, a)$ the number of bacteria lost in this first phase (*i.e.* $\mu(x, a) = 1$ or $\mu(x, a) = \rho(x, a)$).

Let us now turn to the second phase. Using Hubbell's model with some modifications (Hubbell does not include the a-variable), the probabilities that a resistant or a sensitive bacterium reappears are, respectively,

$$Q_r = Q_r(R) = m\bar{s} + (1 - m)s_j, \qquad Q_s = 1 - Q_r, \tag{1}$$

where

$$s_j = \frac{R_j}{J - \mu(R_j, a)}, \quad \text{and} \quad \bar{s} = \frac{1}{N}\sum_{j=1}^{N} \frac{R_j}{J - \mu(R_j, a)}, \tag{2}$$

and $m \in [0, 1]$ is the probability that a bacterium is chosen from the metacommunity as a whole, rather than from the focal individual.

However, since each time step (the two phases) can be arbitrarily small, at least in the mathematical model, we will in this model make the assumption that $\mu(R, a)/J$ is very small (although the number $\mu(R, a)$ can still be large), so that $R/(J - \mu(R, a))$ is very close to R/J. Actually in the main result below, we even assume equality here, so

$$Q_r = m\bar{r} + (1 - m)r_j, \qquad Q_s = 1 - Q_r, \tag{3}$$

where

$$r_j = \frac{R_j}{J}, \quad \text{and} \quad \bar{r} = \frac{1}{N}\sum_{j=1}^{N} r_j. \tag{4}$$

We call $h = 1 - m$ *the hygiene parameter*, so that good hygiene means high h-values and low m-values (and vice versa). This procedure is performed precisely $\mu(R, a)$ times to replace all bacteria lost in the first phase.

Even if we take into account that the number of resistant bacteria after the first phase has changed, when looking at the probabilities Q_r and Q_s, i.e. using (1), the main result will change very little if $J(1 - \varepsilon) \leq (J - \mu(R, a))$ for some small $\varepsilon > 0$. We may also consider $\bar{s}$ as the total portion of resistant bacteria in the whole metacommunity. A similar computation yields the same conclusion. We discuss this in the proof.

If we look at the transition probabilities for all hosts, this creates a huge Markov chain, since the number N of people and the total number J of bacteria are usually very large numbers. We will instead consider the expected change Z of resistant bacteria in the whole population, given an antibiotic pressure a, the level of hygiene h, and the number of resistant bacteria R_j in each host j, $1 \leq j \leq N$. So Z is a function of a, h, and the numbers R_j, $1 \leq j \leq N$.

In the proposition below, we note first that the function $\rho(x, a)$ is in reality integer valued. However, in the proposition it is assumed to satisfy a Lipschitz continuity condition (with differentiability in a). We then take the integer part to go back to its real meaning. When one takes the integer part of ρ, the partial derivatives in the proposition have to be replaced by corresponding "discrete" derivatives.

On the other hand, one can view the death and rebirth processes of bacteria as binomially distributed; the death process $X \sim Bin(\rho/J, J)$ and the rebirth process Y, where $Y|X \sim Bin(Q_r, X)$ and hence $\mathbb{E}(Y|X) = Q_r X$ is the conditional expectation. Then, since $\mathbb{E}(X) = \rho$, we have

$$\mathbb{E}(Y) = \mathbb{E}(\mathbb{E}(Y|X)) = \mathbb{E}(Q_r X) = Q_r \rho.$$

As $J \to \infty$, we note that the death binomial process will converge to a Poisson process.

In the proposition below, $\rho(x, a)$ is considered as a function of the relative abundance x of resistant bacteria, *i.e.*, $x \in [0, 1]$. Write $\frac{\partial \rho}{\partial a} = \partial_a \rho$.

Proposition 1.1. *Given $Z = Z(a, h)$ as above. Suppose that the function $\rho(x, a) \geq 1$ is decreasing in x, $\rho(x, a)$ and $\partial_a \rho(x, a) \geq 0$ are Lipschitz continuous, $\rho(x, 1) = 1$ and that*

$$k(x, a) = x(\rho(x, a) - 1) + \partial_a \rho(x, a) a(a - x) \tag{5}$$

is decreasing in x. Then,

$$\frac{\partial^2 Z}{\partial h \partial a} \leq 0, \tag{6}$$

with strict inequality if $a > 1$.

In the computations we will use the parameter $m = 1 - h$ and hence prove the equivalent statement

$$\frac{\partial^2 Z}{\partial m \partial a} \geq 0. \tag{7}$$

We call this *the first prediction.*

We now discuss the condition that (5) is decreasing in x (we refer to this condition simply as (5)). If for example $\rho(x, a) = \phi(a)h(x) + 1$, where $h(x)$ is decreasing in x then (5) is equivalent to the statement that

$$h(x)(x(\phi(a) - a\phi'(a)) + a^2\phi'(a))$$

is decreasing in x. Since $k(x, a) \geq 0$, this is true in particular if $\phi(a) - a\phi'(a) \leq 0$ which is equivalent to

$$\frac{\partial}{\partial a}\left(\frac{\phi(a)}{a}\right) \geq 0.$$

There are many functions that satisfy this condition (5). A simple example is $\rho(x, a) = 1 + (a - 1)(1 - x)$. We will also write $\rho'_a(x, a) = \frac{\partial \rho}{\partial a}(x, a)$.

As a by–product we have:

The greater the β–diversity of the metacommunity, the smaller the competitive release of resistant bacteria in response to antibiotic pressure.

We call this the *second prediction.*

2. Proof of Proposition 1.1

The aim in this section is to study the expected change in the number of resistant bacteria in the whole population, given certain values on a (antibiotic pressure) and m (hygiene). Also, we will see that there is an interplay between the β–diversity of the metacommunity and its response to antibiotic pressure.

In the first phase, a number of bacteria are lost; one resistant with probability P_r or ρ sensitive with probability $P_s = 1 - P_r$. In order to have a zero sum process, all bacteria lost in the first phase must be replaced by new ones in the second phase. So if ρ sensitive bacteria were lost in the first phase (in some host), then obviously ρ new bacteria must be reborn, and if a resistant bacterium was lost in the first phase, we only need to add one extra bacterium in the second phase.

We proceed as in Hubbell's model, but perform the bacterial replacement procedure ρ times if sensitive bacteria were lost in the first phase, and only once if a resistant bacterium was lost in the first phase. Recall $Q_r = Q_r(R_j)$ and $Q_s = Q_s(R_j)$ for the probabilities for host j that one resistant or sensitive bacterium reappears, respectively.

We let X be the total number of resistant bacteria lost in the first phase and Y be the total number of resistant bacteria that reappear in the second phase. Moreover, let X_j be the number of resistant bacteria lost for host individual j in the first phase, and Y_j the number of reappearing resistant bacteria for host individual j in the second phase. So we have

$$X = \sum_j X_j, \quad X_j \in \{0, 1\}$$
$$Y = \sum_j Y_j, \quad Y_j \in \{0, \dots, \rho\},$$

where $\rho = \rho(R_j, a)$. We then put

$$Z = \mathbb{E}(Y - X) \quad \text{and} \quad Z_j = \mathbb{E}(Y_j - X_j).$$

Then Z is the expected total change of resistant bacteria after one full time step (both phase one and phase two) and

$$Z = \mathbb{E}(Y - X) = \mathbb{E}(Y) - \mathbb{E}(X) = \sum_j \mathbb{E}(Y_j) - \sum_j \mathbb{E}(X_j). \tag{8}$$

To compute $\mathbb{E}(X_j)$ is easy. If we let $r_j = R_j/J$, we get

$$\mathbb{E}(X_j) = \frac{r_j}{a} \cdot 1 + (1 - \frac{r_j}{a}) \cdot 0 = \frac{r_j}{a}.$$

To compute $\mathbb{E}(Y_j)$ is more complicated since they depend on X_k, $1 \le k \le N$. Let s_j be the portion of resistant bacteria after the first phase, so that $s_j = r_j \frac{1}{1-\mu(r_j,a)/J}$. If a resistant bacterium was lost in the first phase (*i.e.*, $X_j = 1$), then the expectation of the change of resistant bacteria in phase two is (recall the probabilities Q_r for picking a resistant and $1 - Q_r$ for a sensitive bacterium, according to (1))

$$\begin{aligned}\mathbb{E}(Y_j | X_1, \dots, X_N, X_j = 1) &= Q_r(R_j) \cdot 1 + Q_s(R_j) \cdot 0 \\ &= (m\bar{s}(X_1, \dots, X_N) + (1-m)s_j) \cdot 1 \\ &\quad + (m(1 - \bar{s}(X_1, \dots, X_N)) + (1-m)(1 - s_j)) \cdot 0 \\ &= m\bar{s}(X_1, \dots, X_N) + (1-m)s_j, \end{aligned} \tag{9}$$

where $\bar{s} = \bar{s}(X_1, \dots, X_N)$ is the average of all s_j, *i.e.*, the average of the portions of resistant bacteria in each host *after* the first phase (and $X_j = 1$). If sensitive bacteria were lost in the first phase, then perform the growth process as above but $\rho(R_j, a)$ times and consequently

$$\begin{aligned}\mathbb{E}(Y_j | X_1, \dots, X_N, X_j = 0) &= \sum_{k=1}^{\rho(r_j)} \big(Q_r(R_j) \cdot 1 + Q_s(R_j) \cdot 0\big) \\ &= \rho(R_j, a)(m\bar{s}(X_1, \dots, X_N) + (1-m)s_j), \end{aligned} \tag{10}$$

where $X_j = 0$. Now, $\bar{s}(X_1, \dots, X_N)$ can change quite drastically if a lot of sensitive bacteria were lost in the first phase, but not if resistant bacteria were lost in the first phase. However, we assume that the portion of resistant bacteria does not change too much after the first phase, which is equivalent to saying that the quotient ρ/J is very small, which is the assumption which was mentioned before, see (3). However, the outcome of Y_j still depends drastically on X_j. Let us write $\rho(R_j, a) = \rho(r_j, a)$ to

simplify the notation in the coming computations, so that we regard $\rho(r_j, a)$ also as a function of the relative abundance of resistant bacteria for individual j. We have

$$\begin{aligned} &\mathbb{E}(Y_j|X_j = 1) = (m\bar{s} + (1-m)s_j), \\ (11) \qquad &\mathbb{E}(Y_j|X_j = 0) = \rho(r_j, a)(m\bar{s} + (1-m)s_j). \end{aligned}$$

The approximation to replace r_j with s_j means that that $\mathbb{E}(Y_j|X_j = 1)$ and $\mathbb{E}(Y_j|X_j = 0)$ are comparable to $m\bar{r} + (1-m)r_j$ and $\rho(r_j, a)(m\bar{r} + (1-m)r_j)$, respectively. To simplify notations, we write $x \sim_\varepsilon y$ if x and y are comparable in this sense, *i.e.*,

$$x \le y \le \frac{1}{1-\varepsilon} x.$$

We now get

$$\begin{aligned} \mathbb{E}(Y_j) &= \mathbb{E}(Y_j|X_j = 1)P(X_j = 1) + \mathbb{E}(Y_j|X_j = 0)P(X_j = 0) \\ &= \frac{r_j}{a}(m\bar{s} + (1-m)s_j) + (1 - \frac{r_j}{a})\rho(r_j, a)(m\bar{s} + (1-m)s_j) \\ (12) \qquad &= m(\frac{r_j}{a} + (1 - \frac{r_j}{a})\rho(r_j, a))(\bar{s} - s_j) + \left(\frac{r_j}{a} + (1 - \frac{r_j}{a})\rho(r_j, a)\right)s_j. \end{aligned}$$

Consequently,

$$\begin{aligned} \mathbb{E}(Y_j - X_j) &= \mathbb{E}(Y_j) - \mathbb{E}(X_j) \\ (13) \qquad &= m(\frac{r_j}{a} + (1 - \frac{r_j}{a})\rho(r_j, a))(\bar{s} - s_j) + \left(\frac{r_j}{a} + (1 - \frac{r_j}{a})\rho(r_j, a)\right)s_j - \frac{r_j}{a}. \end{aligned}$$

So, regarding s_j as a function of r_j,

$$Z_j = \mathbb{E}(Y_j - X_j) = m\beta(r_j, a)(\bar{s} - s_j) + \alpha(r_j, a) \sim_\varepsilon m\beta(r_j, a)(\bar{r} - r_j) + \alpha(r_j, a),$$

where

$$(14) \qquad \beta(r_j, a) = \frac{r_j}{a} + (1 - \frac{r_j}{a})\rho(r_j, a), \text{ and}$$

$$(15) \qquad \alpha(r_j, s_j, a) = \left((1 - \frac{r_j}{a})\rho(r_j, a) + \frac{r_j}{a}\right)s_j - \frac{r_j}{a},$$

and where we have set $\alpha(r_j, r_j, a) = \alpha(r_j, a)$. Note that r does not depend on the parameter a (antibiotic pressure), but s does.

We want to show that $\partial Z_j / \partial a$ increases in m, *i.e.*, that $\partial^2 Z_j / \partial m \partial a \ge 0$. To do this, we will first need that $\beta'_a(x) = \partial\beta/\partial a(x)$ is positive and decreasing in x. We have

$$(16) \qquad \beta'_a(r_j) = \frac{r_j}{a^2}(\rho(r_j, a) - 1) + \rho'_a(r_j, a)(1 - \frac{r_j}{a}).$$

Since $\rho'_a(x, a)$ is positive from the assumptions and $\rho(r_j, a) \ge 1$, we clearly have $\beta'(a) \ge 0$. We may without loss of generality order the r_j so that $r_1 \le r_2 \le \ldots \le r_N$. Recall that both ρ and ρ'_a are decreasing functions in x. The condition (5) means that $\beta'_a(x)$ is decreasing in x and hence $\beta'_a(r_j, a)$ is decreasing in j. To prove (7), we need the following lemma, which we state in a more general form.

Lemma 2.1. *Let $\xi \ge 0$ be a decreasing function and $f \ge 0$, both defined on a compact interval I. Suppose that $\mu(I) = 1$. Then*

$$\int_I \xi \circ f(x)\, \mathrm{d}\mu \int_I f(x)\, \mathrm{d}\mu \ge \int_I \xi \circ f(x) f(x)\, \mathrm{d}\mu.$$

T. Persson pointed out that this lemma is quite similar to the Chebyshev integral inequality and supplied an elegant proof based on this. However, for our purposes we present another proof.

Proof. Let $\alpha(x) = f(x) - \int f(x)\,\mathrm{d}\mu$. Then $\int \alpha(x)\,\mathrm{d}\mu = 0$. Put $A = \{x : \alpha(x) \geq 0\}$ and $B = \{x : \alpha(x) < 0\}$. Since $\xi(x)$ is decreasing, we have

$$
\begin{aligned}
\int \xi \circ f(x) \Big(f(x) - \int f(x)\,\mathrm{d}\mu \Big)\,\mathrm{d}\mu &= \int \xi(f(x))\alpha(x)\,\mathrm{d}\mu \\
&= \int_A \xi(f(x))|\alpha(x)|\,\mathrm{d}\mu - \int_B \xi(f(x))|\alpha(x)|\,\mathrm{d}\mu \qquad (17)\\
&\leq \int_A \xi\Big(\int f(x)\,\mathrm{d}\mu \Big)|\alpha(x)|\,\mathrm{d}\mu - \int_B \xi\Big(\int f(x)\,\mathrm{d}\mu \Big)|\alpha(x)|\,\mathrm{d}\mu \qquad (18)\\
&= \xi\Big(\int f(x)\,\mathrm{d}\mu \Big) \int \alpha(x)\,\mathrm{d}\mu = 0.
\end{aligned}
$$

This proves the lemma. □

Remark 2.2. It also follows from the proof of the lemma that there are constants $c, c' \geq 0$ which only depend on ξ such that

$$
\begin{aligned}
c \int_I \Big| f(x) - \int_I f(x)\,\mathrm{d}\mu \Big|^2 \mathrm{d}\mu &\leq \int_I \xi \circ f(x)\,\mathrm{d}\mu \int_I f(x)\,\mathrm{d}\mu - \int_I \xi \circ f(x) f(x)\,\mathrm{d}\mu \\
&\leq c' \int_I \Big| f(x) - \int_I f(x)\,\mathrm{d}\mu \Big|^2 \mathrm{d}\mu. \qquad (19)
\end{aligned}
$$

Indeed, the difference between the first terms on the lines (17) and (18) is

$$
\begin{aligned}
&\int_A \xi\Big(\int f(x)\,\mathrm{d}\mu \Big)|\alpha(x)|\,\mathrm{d}\mu - \int_A \xi(f(x))|\alpha(x)|\,\mathrm{d}\mu \\
&\qquad = \int_A \Big(\xi\Big(\int f(x)\,\mathrm{d}\mu \Big) - \xi(f(x)) \Big)|\alpha(x)|\,\mathrm{d}\mu.
\end{aligned}
$$

Since ξ is Lipschitz continuous, and since $\alpha(x) \geq 0$ on A, we conclude that there are constants $c_1 \geq 0$ and $c_2 \geq 0$ that only depend on ξ such that

$$
\begin{aligned}
c_1 \int_A |\alpha(x)|^2\,\mathrm{d}\mu &\leq \int_A \Big(\xi\Big(\int f(x)\,\mathrm{d}\mu \Big) - \xi(f(x)) \Big)|\alpha(x)|\,\mathrm{d}\mu \qquad (20)\\
&\leq c_2 \int_A |\alpha(x)|^2\,\mathrm{d}\mu.
\end{aligned}
$$

A similar inequality holds for the difference of the two last terms in (17) and (18). Indeed, by the same argument, remembering that $\alpha(x) \leq 0$ on B, there are constants $d_1 \geq 0$ and $d_2 \geq 0$ such that

$$
\begin{aligned}
d_1 \int_B |\alpha(x)|^2\,\mathrm{d}\mu &\leq \int_A \Big(\xi(f(x)) - \xi\Big(\int f(x)\,\mathrm{d}\mu \Big) \Big)|\alpha(x)|\,\mathrm{d}\mu \qquad (21)\\
&\leq d_2 \int_B |\alpha(x)|^2\,\mathrm{d}\mu.
\end{aligned}
$$

So we see that

$$c_1 \int_A |\alpha(x)|^2 \,\mathrm{d}\mu + d_1 \int_B |\alpha(x)|^2 \,\mathrm{d}\mu$$
$$\leq \left| \int_I \xi \circ f(x) \,\mathrm{d}\mu \int_I f(x) \,\mathrm{d}\mu - \int_I \xi \circ f(x) f(x) \,\mathrm{d}\mu \right|$$
$$\leq c_2 \int_A |\alpha(x)|^2 \,\mathrm{d}\mu + d_2 \int_B |\alpha(x)|^2 \,\mathrm{d}\mu.$$

Taking $c = \min(c_1, d_1)$ and $c' = \max(c_2, d_2)$ gives the desired result.

Recall that $Z = \sum_j Z_j$ can be written as

$$Z = Bm + A,$$

where

$$B = \sum_j (\bar{s} - s_j)\beta(r_j, a) \quad \text{and} \quad A = \sum_j \alpha(r_j, a)$$

both depend on a. Therefore, with $A'_a = \sum_j \alpha'_a(r_j, a)$, we have

$$\frac{\partial Z}{\partial a} = mB'_a + A'_a,$$

and

$$\frac{\partial^2 Z}{\partial m \partial a} = B'_a = \sum_{j=1}^{N} \frac{\partial}{\partial a}\Big((\bar{s} - s_j)\beta(r_j, a) \Big) \tag{22}$$

$$= \sum_{j=1}^{N} \beta(r_j, a) \frac{\partial}{\partial a}(\bar{s} - s_j) + \beta'_a(r_j, a)(\bar{s} - s_j). \tag{23}$$

We want to argue that the second term in the last sum is dominant. The only interesting case is when $\mu(r_j, a) = \rho(r_j, a)$ so suppose that $s_j = r_j(1 - \rho(r_j, a)/J)$. Fix two indices i and j and set $s_i = r_i/(1 - \rho(r_i, a)/J)$. Then

$$\frac{\partial}{\partial a} s_i \beta(r_j, a) = \beta'_a(r_j, a) \left(s_i + \frac{r_i \rho'_a(r_i, a)}{J(1 - \rho(r_i, a)/J)^2} \frac{\beta(r_j, a)}{\beta'_a(r_j, a)} \right).$$

Recalling the expressions for β and β'_a, we have

$$\frac{\beta(r_j, a)}{\beta'_a(r_j, a)} \leq \frac{\rho(r_j, a)}{(1 - r_j/a)\rho'_a(r_j, a)},$$

so

$$\frac{r_i \rho'_a(r_i, a)}{J(1 - \rho(r_i, a)/J)^2} \frac{\beta(r_j, a)}{\beta'_a(r_j, a)} \leq \frac{r_i \rho(r_j, a)}{J(1 - \varepsilon)^2 (1 - r_j/a)} \frac{\rho'_a(r_i, a)}{\rho'_a(r_j, a)}. \tag{24}$$

Here we need some kind of bound on the distortion of $\rho(r, a)$ in the a-derivative, *i.e.*, that $\rho'_a(r, a)$ does not change too much in the following sense; there is some constant $C \geq 1$ such that

$$\frac{1}{C} \leq \frac{\rho'_a(r, a)}{\rho'_a(s, a)} \leq C,$$

for all $r, s \in [0, 1]$ and all a-values which are realistic, that is, when a belongs to some interval $[1, A]$. This condition on ρ is quite natural, if we impose some regularity on ρ and have in mind that $\rho(r, a) \leq \varepsilon J$, for all $r \in [0, 1]$ and $a \in [1, A]$, for some fixed (very) small $\varepsilon > 0$.

If we have such a distortion estimate, then the last term in (24) is a very small portion of r_i if $1 - r_i/a \geq 1 - r_i \geq \delta > 0$ for some δ (depending on J), so that $C\rho(r_j, a)/\delta J$ still is very small. So choosing J sufficiently large, we get

$$\frac{\partial^2 Z}{\partial m \partial a} \sim_{\varepsilon'} \sum_{j=1}^{N} (\bar{s} - s_j)\beta'_a(r_j, a) \sum_{j=1}^{N} \sim_{\varepsilon'} (\bar{r} - r_j)\beta'_a(r_j, a), \tag{25}$$

for some small ε'. Hence if, for example, $1 - r \geq \delta$, even if we take into account that the probability that resistant (or sensitive) bacteria reappear in the second phase depends on the number of resistant bacteria *after* the first phase, it will make a very small difference to the main result above. The condition $1-r \geq \delta$ can be made weaker the larger J is. Many examples of $\rho(r, a)$, for instance $\rho(r, a) = 1 + (a - 1)(1 - r)$, fits into this picture. With minor changes, a similar result can be obtained if $\bar{s}$ is the total portion of resistant bacteria in the whole metacommunity. We leave the details to the reader.

Applying the lemma to the uniformly distributed point measure $\mu = \frac{1}{N} \sum_{j=1}^{N} \delta_j(x)$ on the interval $I = [1, N]$, where $f(j) = r_j$ and $\xi(x) = \beta'_a(x)$ it follows that

$$B'_a = \frac{\partial B}{\partial a} = \sum_{j=1}^{N} (\bar{r} - r_j)\beta'_a(r_j, a) \geq 0,$$

with strict inequality if $a > 1$ and the distribution of r_j is such that not all are equal. In particular, if the β-diversity is zero, *i.e.,* the distribution of the resistant bacteria is completely uniform, then $B'_a = 0$. This proves our first prediction (Proposition 1.1).

We also note that $\partial Z/\partial a$ is clearly positive since every $\alpha'_a(r_j, a) \geq 0$, $1 \leq j \leq N$ (this is simply equivalent to the unsurprising fact that the greater the antibiotic use in the population, the faster the proportion of bacteria that are resistant increases).

From the remark after the lemma, putting $\alpha(j) = \bar{r} - r_j$, we see that the sum B'_a is comparable to the deviation:

$$B'_a \sim \sum_j |\bar{r} - r_j|^2.$$

Hence, we have also settled the second prediction.

Remark 2.3. A natural question is to ask whether

$$B = \frac{\partial Z}{\partial m} = \sum_{j=1}^{N} (\bar{r} - r_j)\beta(r_j, a) \geq 0.$$

But this follows easily from the fact that $\partial^2 Z/\partial m \partial a \geq 0$ and integrating. Suppose that $m_1 \leq m_2$ and fix some $a \geq 1$. Then

$$Z(m_1, a) = \int_1^a \partial_a Z(m_1, t)\, \mathrm{d}t, \tag{26}$$

$$Z(m_2, a) = \int_1^a \partial_a Z(m_2, t)\, \mathrm{d}t. \tag{27}$$

Since $\partial_a Z$ is increasing in m, we have $Z(m_1, a) \leq Z(m_2, a)$ for all $a \geq 1$ and all choices of $m_1 \leq m_2$. Hence, $B \geq 0$.

Remark 2.4. Another consequence of the model is that if no antibiotics are present, then Z will no longer depend on m. Indeed, since $\rho(x,1)=1$, we have

$$B=\sum_j \beta(r_j,1)(\bar{r}-r_j)=\sum_j \bar{r}-r_j=0.$$

Hence, hygiene has no effect on the rate of increase of the number of resistant bacteria in the absence of antibiotics.

Analysis of a simple compartmental model

It is common to study the spread of antibiotic resistant bacteria using the epidemiological compartmental modelling framework (2), and to connect our results to this rich tradition, we here show the main result of our study within this framework. The within-host diversity of bacteria is modelled as a subdivision of the host population into compartments with different portions of different bacterial strains. Since this is necessarily a crude representation of diversity, we will opt for simplicity rather than nuance in the choice of model. We begin with a model with 4 compartments, and then simplify.

Consider a commensal that is ubiquitous, or nearly so, such as *E. coli.* There are two strains, a sensitive strain and a resistant strain. And hosts are divided into compartments based on which strain(s) they harbour. Hosts in compartment S have only the sensitive strain, and those in R only the resistant. Hosts in compartment S_R harbour mostly sensitive bacteria, but there are a few resistant cells, and hosts in R_S have mostly resistant bacteria, but there are a few sensitive cells. Hosts flow from S to S_R as they contract resistant bacteria by transmission. From S_R they can either return to S, as the sensitive bacteria outcompete the resistant bacteria due to a fitness cost of resistance, or continue to R due to competitive release under antibiotic treatment that kills the sensitive cells. From R, hosts flow to R_S by transmission of sensitive bacteria, and from there either return to R due to treatment, or transition to S due to a cost of resistance. The model is represented in *Figure 3 A*.

For simplicity, now assume that the cost of resistance is negligible, such that there are no flows from S_R to S or from R_S to S. The resulting model is represented in *Figure 3 B.* Since hosts in R and R_S cannot leave this pair of compartments, and R_S is dominated by resistant bacteria, R and R_S can be combined. This yields the model represented in *Figure 3 C.* This model is described by the following ordinary differential equations:

$$\frac{dR}{dt}=aS_R, \tag{28}$$

$$\frac{dS_R}{dt}=\beta SR-aS_R, \tag{29}$$

$$\frac{dS}{dt}=-\beta SR, \tag{30}$$

$$S+R+S_R=1, \tag{31}$$

where a is the antibiotic pressure on the host population and β is the transmission rate. Improvements in hygiene lower β. Competitive release under antibiotic treatment is represented by the flow from S_R to R according to aS_R.

Let us first discuss the model informally to build intuition. The first prediction, that hygiene should attenuate the effect of antibiotic pressure on the competitive release of resistant bacteria, can be intuited by noting, firstly, that the rate at which

R increases in the model in *Figure 3 C* depends on both the antibiotic pressure, a, and the proportion of hosts that are in compartment S_R, and, secondly, that the rate at which new hosts enter compartment S_R, and thus become available to treatment induced competitive release, depends on β, which, in turn, decreases with hygiene. The second prediction, that this is because improvements in hygiene make resistant strains less uniformly distributed across the host population, can also be intuited from the model. Note, firstly, that the distribution of resistant strains is most uniform when S_R is large, and, secondly, that the rate of increase in R depends on S_R. Put another way, a large S_R corresponds to the situation discussed in the main text, where resistant bacteria are present in many host individuals, but have a low abundance in the hosts in which they are present.

We now give a sketch of a proof that the first (and main) prediction follows from this model, at least under some circumstances (conditions on the prevalences of R and S_R). We stress that this prediction holds *locally around any given initial values on* R *and* S_R. With some conditions on R and S_R, we can prove this globally as well, meaning far away from the initial values. These conditions can most likely be relaxed, at least up to some point, but for our purposes we only wish to illustrate that the same phenomenon can be found in this compartment model as in the metacommunity model.

Assume that the system of differential equations above is defined for $t \geq 0$ with some initial values $S_R(0)$ and $R(0)$. The first prediction is equivalent to the proposition that the second partial derivative of R satisfies

$$\frac{\partial^2 R(t)}{\partial a \partial \beta} \geq 0, \tag{32}$$

with strict inequality for $t > 0$. Locally, *i.e.*, for t close to 0 this is always true, as we will see below.

Let us first reduce the equations to a system in two variables, in R and S_R. Let us also use the simpler variables $x = R$ and $y = S_R$:

$$\frac{dy}{dt} = \beta(1 - y - x)x - ay, \tag{33}$$

$$\frac{dx}{dt} = ay. \tag{34}$$

By making a simple change of variables (e.g. $\hat{y} = y/\beta$, $\hat{x} = x$) one can easily see that (32) is satisfied locally around any initial point $y_0 = y(x_0)$, where $x_0 = x(0)$ and $y_0 = y(0)$, (note that the initial point is not dependent on the parameters a and β). However, in order to analyse the long term behaviour, we take another route.

Eliminating the t-variable, we get

$$\frac{dy}{dx} = y' = \frac{\beta}{a}(1 - y - x)\frac{x}{y} - 1 = \frac{\beta}{a}(1 - x)\frac{x}{y} - \frac{\beta}{a}x - 1. \tag{35}$$

Hence, the solution curves $y = y(x)$ only depend on $\gamma = \frac{\beta}{a}$ and the initial value $y(x_0) = y_0$. So let us keep in mind that $y = y(x, \gamma)$, and write

$$y' = \gamma(1 - x)\frac{x}{y} - \gamma x - 1.$$

We also note that $R' = x' = ay(x, \gamma)$, and thus, after some calculations,

$$\frac{\partial^2 R'}{\partial a \partial \beta} = -\frac{\beta}{a^2}\frac{\partial^2 y}{\partial \gamma^2}.$$

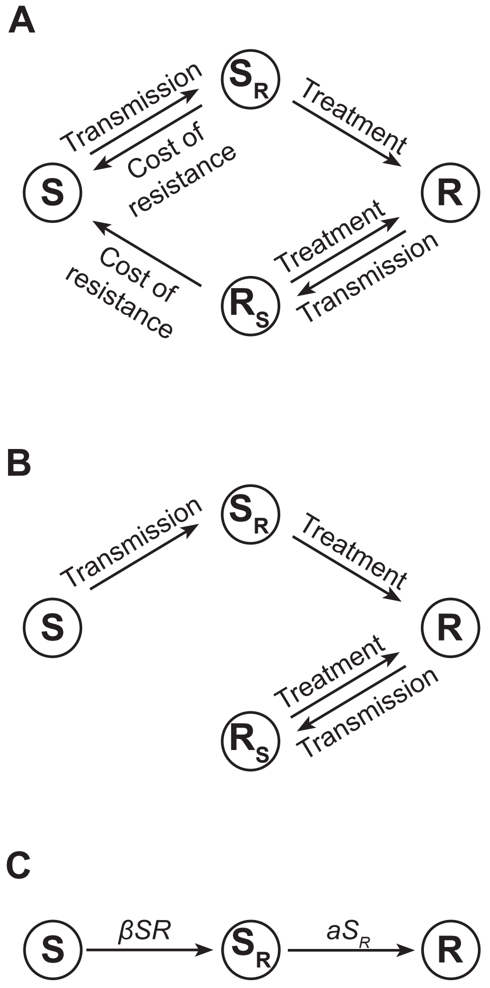

FIGURE 1

We recall that both a and β are positive numbers. So if we can show that $y_{\gamma\gamma} = \frac{\partial^2 y}{\partial \gamma^2}$ is negative, the result follows by integrating in the time-domain.

We first show that $y_\gamma = \frac{\partial y}{\partial \gamma}$ is non-negative. Differentiating (35) with respect to γ we get,

$$y'_\gamma = (1-x)\frac{x}{y} - \gamma(1-x)x\frac{y_\gamma}{y^2} - x. \tag{36}$$

Considering this as a linear equation in y_γ, assuming that a solution $y = y(x, \gamma)$ exists, we write

$$y'_\gamma + \gamma(1-x)x\frac{y_\gamma}{y^2} = (1-x)\frac{x}{y} - x. \tag{37}$$

We see that the integrating factor

$$\mu(x) = \exp\left\{\int_{x_0}^{x} \gamma(1-\tau)\frac{\tau}{y^2}\,\mathrm{d}\tau\right\}$$

satisfies $\mu(x) \geq 1$ and is increasing. We get, after integrating,

$$y_\gamma = \frac{1}{\mu(x)} \int_{x_0}^{x} \mu(\xi) \frac{\xi}{y}(1 - \xi - y)\,\mathrm{d}\xi.$$

Since $\mu(x) \geq 1$ and the integrand is non-negative, we have $y_\gamma \geq 0$.

We now go one step further to investigate the sign of $y_{\gamma\gamma}$. Differentiating (37) with respect to γ we get

$$y'_{\gamma\gamma} = -2(1-x)x\frac{y_\gamma}{y^2} - \gamma(1-x)x\frac{y_{\gamma\gamma}}{y^2} + 2\gamma(1-x)x\frac{y_\gamma^2}{y^3}, \tag{38}$$

which is equivalent to

$$y'_{\gamma\gamma} + \gamma(1-x)\frac{x}{y^2}y_{\gamma\gamma} = -2(1-x)x\frac{y_\gamma}{y^3}(y - y_\gamma). \tag{39}$$

Multiplying by the same integrating factor $\mu(x)$, and integrating we see that $y_{\gamma\gamma}$ is non-positive if $y - y_\gamma \geq 0$.

We see directly that *locally* we have $y_{\gamma\gamma} \leq 0$, since $y_\gamma(x_0) = 0$ (the starting point does not depend on γ).

In forward time (when $y = y(x)$ is further away from the initial value $y_0 = y(x_0)$) it is also true under some circumstances, *i.e.*, conditions on x and y, and possibly it is simply true for all $0 \leq x, y \leq 1$. We will not go through all possible such conditions on y and x here, but as an example, suppose that $y \geq x$. Then, since $\mu(x)$ is increasing, by the Mean Value Theorem for integrals, for some $0 \leq \eta \leq x$,

$$y_\gamma = \frac{\mu(\eta)}{\mu(x)} \int_{x_0}^{x} \frac{\xi}{y}(1 - \xi - y)\,\mathrm{d}\xi \leq \int_{x_0}^{x} \frac{\xi}{y}(1 - \xi - y)\,\mathrm{d}\xi. \tag{40}$$

Using that $\xi \leq y$ we get

$$y_\gamma \leq \int_{x_0}^{x} \frac{\xi}{y}(1 - \xi - y)\,\mathrm{d}\xi \leq \int_{x_0}^{x} (1 - \xi - y)\,\mathrm{d}\xi \leq x - x_0 \leq x \leq y. \tag{41}$$

So as long as $y \geq x$ the statement holds that $y_{\gamma\gamma} \leq 0$ (with strict inequality if $x, y \neq 0, 1$). If we require that $y \geq \min(x, 1-x)$, which is a slightly stronger condition, then, using that $y \geq x$ on $[0, 1/2]$ and that $y \geq 1 - x$ on $[1/2, 1]$,

$$\begin{aligned}
y_\gamma &\leq \int_{x_0}^{x} \frac{\xi}{y}(1 - \xi - y)\,\mathrm{d}\xi \\
&= \int_{[x_0,x]\cap[0,1/2]} \frac{\xi}{y}(1 - \xi - y)\,\mathrm{d}\xi + \int_{[x_0,x]\cap[1/2,1]} \frac{\xi}{y}(1 - \xi - y)\,\mathrm{d}\xi \\
&\leq \int_{[x_0,x]\cap[0,1/2]} (1 - 2\xi)\,\mathrm{d}\xi + \int_{[x_0,x]\cap[1/2,1]} 0\,\mathrm{d}\xi \\
&\leq \int_{[x_0,x]\cap[0,1/2]} (1 - 2\xi)\,\mathrm{d}\xi \leq \frac{1}{2} - x_0 - (\frac{1}{4} - x_0^2) \leq \frac{1}{4}.
\end{aligned} \tag{42}$$

Combining this with the estimate just before (see (41)), this means that $y_\gamma \leq y$ in the almost triangle shaped domain $D = \{(x, y) : y \geq \min(x, 1-x)\mathcal{X}_{[0,3/4]} + \frac{1}{4}\mathcal{X}_{[3/4,1]}\}$, where $\mathcal{X}_I$ is the characteristic function on I (*i.e.*, the function that takes the value 1 on I and 0 elsewhere). Although it is very likely that these estimates can be improved, they show that the phenomenon we proved in the metacommunity model also holds in this simple compartment model, at least for a large proportion of the states in the phase space. Finally, we recall that the phenomenon holds locally

around every initial point. This does not imply a global statement, since far away from these initial values, $x = R$ and $y = S_R$ may take different routes for different parameters a and β.

Supplement B: Statistical analysis of resistance data

3. Introduction

Here we analyse data on antibiotic resistance, antibiotic use, and consumption of alcohol-based hand rub in long-term care facilities (LTCFs) given in the report "Point prevalence survey of healthcare-associated infections and antimicrobial use in European long-term care facilities. April-May 2013" from the European Centre for Disease Prevention and Control (ECDC), henceforth "the LTCF report" (3). We also use data on nation-wide resistance from the ECDC surveillance report "Antimicrobial resistance surveillance in Europe 2014" (4). The analyses were performed in R version 3.5.0 (5).

4. Description of the data

The dataset analysed includes for LTCFs in each country the median consumption of alcohol-based hand rub in litres per 1000 resident days, the percentage of residents that were being treated with antibiotics at the time point when the study was performed, the percentage of treated residents receiving penicillins, the percentage receiving other β-lactams, the proportion of *Enterobacteriaceae* isolates from the facilities that were resistant to third generation cephalosporins, and the number of *Enterobacteriaceae* isolates that were tested for resistance. It also includes for each country the proportion of *E. coli* isolates that were resistant to third generation cephalosporins in nation-wide surveillance (not only LTCFs) in the same year. The hand rub data (*handrub*) were extracted from the map in Figure 17 in the LTCF report, and were coded as the centre of the given interval. Data given as ≥ 8 were coded as 10. The percentage of residents on antibiotic treatment was taken as the medians from Table 17 in the LTCF report, and the percentages of treated residents on different antibiotics (penicillins and other β-lactams, respectively) were taken from Table 19 in the LTCF report. The data on *Enterobacteriaceae* resistant to third generation cephalosporins were extracted by measurement in Figure 36 in the LTCF report, using Adobe Photoshop, and proportions were calculated for the isolates for which the resistance status was known. These proportions were then multiplied with the number of isolates tested, to yield the counts of resistant and sensitive isolates. The proportion of *E. coli* resistant to third generation cephalosporins in nation-wide surveillance pertain to 2013, and were taken from Table 3.4 in the ECDC report "Antimicrobial resistance surveillance in Europe 2014". Only countries that provided data for all variables were included in the analysis.

5. Statistical analyses

The log-odds of resistance to third generation cephalosporins in *Enterobacteriaceae* was modelled using logistic regression on the counts of resistant and sensitive isolates for each country. Two models were evaluated.

Model 1. The first model is that this log-odds of resistance in the LTCFs is

$$(43) \quad Logit(LTCF) = \beta_0 + \beta_1 \cdot country + \beta_2 \cdot handrub + \beta_3 \cdot \beta\_lactams + \beta_4 \cdot handrub \cdot \beta\_lactams,$$

where *country* is the log-odds of *E. coli* resistant to third generation cephalosporins in each respective country as a whole, *handrub* is the use of alcohol-based hand rub in the LTCFs, and *β_lactams* is the prevalence of β-lactam (penicillins + other β-lactams) treatment in the LTCFs in each country. The inclusion of the resistance in each country as a baseline (*country*) means that the model estimates the effect of the use of hand rub and β-lactams in LCTFs as being proportional to the resistance in the corresponding country. Were we to estimate the difference in the log-odds, we would need to put β_1 exactly equal to 1 in front of the resistance in each country, as this would yield a difference between LTCFs and the country as a whole. (β_0 can be seen as a correction for the difference between *country* and LTCFs.) This model posits that

$$\begin{aligned} Odds(LTCF) &= Odds(country) \cdot \\ &\quad exp(\beta_0 + \beta_2 \cdot handrub + \beta_3 \cdot \beta\_lactams + \beta_4 \cdot handrub \cdot \beta\_lactams). \end{aligned}$$

This is entirely feasible using an offset in the statistical software R. However, there is no strong prior reason to constrain β_1 to be exactly 1. We therefore use *model 1* as stated above (see (43)), thus positing that

$$\begin{aligned} Odds(LTCF) &= Odds(country)^{\beta_1} \cdot \\ &\quad exp(\beta_0 + \beta_2 \cdot handrub + \beta_3 \cdot \beta\_lactams + \beta_4 \cdot handrub \cdot \beta\_lactams), \end{aligned}$$

and retain this model if it gives reasonable output. We thus estimate the effect of hand rub use and β-lactam treatment in the LTCFs on the enrichment of resistant strains in the LTCFs as compared to the general situation in each respective country in which the LTCFs are located, whilst not constraining β_1 to be exactly 1.

The deviance table for this model is:

	Df	Deviance	Resid-Df	Resid-Dev	$\Pr(> \mathcal{X})$
NULL			11	35.9	
country	1	15.9	10	20.0	$6.65 \cdot 10^{-5}$ ***
handrub	1	4.03	9	16.0	0.0448 *
β_lactams	1	3.16	8	12.8	0.0756
handrub · *β_lactams*	1	7.90	7	4.94	0.00495 **

And the table of estimates is:

	Estimate	Lower CI95	Upper CI95
β_0 (Intercept)	-0.649	-1.900	0.5830
β_1 (*country*)	0.932	0.273	1.6600
β_2 (*handrub*)	0.220	-0.089	0.5330
β_3 (*β_lactams*)	0.971	0.386	1.5900
β_4 (*handrub* · *β_lactams*)	-0.157	-0.273	-0.0466

There is thus a significant negative interaction between *handrub* and *β_lactams*. The p-value for the interaction term is well below 0.05, and its CI95 does not include 0.

This model has 5 degrees of freedom and an AIC of 43.2.

Model 2. Since it is biologically unclear whether all β-lactams or only non-penicillin β-lactams should be included, we also use a model with only non-penicillin β-lactams. This model is:

$$\begin{aligned} Logit(LTCF) &= \beta_0 + \beta_1 \cdot country + \beta_2 \cdot handrub + \\ &\quad \beta_3 \cdot other\_\beta\_lactams + \beta_4 \cdot handrub \cdot other\_\beta\_lactams, \end{aligned}$$

where $other\_\beta\_lactams$ includes only non-penicillin β-lactams, and the other variables are defined as above. This model has 5 degrees of freedom and an AIC of 49.6.

Model choice. We retain *model 1*, as it has the lower AIC.

Comments. The dataset has few observations, so if we were to consider confounding factors, a non-significant effect would not be evidence of lack of influence. Since the data are from an observational study, it is impossible to state that the model is correct. However, we believe that it contains the minimal set of explanatory variables.

References

[1] S. P. Hubbell, *The unified neutral theory of biodiversity and biogeography (MPB-32).* Princeton University Press, 2001.

[2] F. Blanquart, "Evolutionary epidemiology models to predict the dynamics of antibiotic resistance," *Evolutionary Applications (doi:10.1111/eva.12753).*

[3] European Centre for Disease Prevention and Control (ECDC), *Point prevalence survey of healthcare-associated infections and antimicrobial use in European long-term care facilities. April-May 2013.* 2014.

[4] European Centre for Disease Prevention and Control (ECDC), *Antimicrobial resistance surveillance in Europe 2014.* 2015.

[5] R. Core Team, "R: A language and environment for statistical computing," *R Foundation for Statistical Computing, Vienna, Austria. URL https://www.R-project.org/.*, 2018.
